# Assessing stigma and perceptions of respectful care in Malawi’s index case testing program: Findings from a cluster randomized controlled trial

**DOI:** 10.1371/journal.pone.0345705

**Published:** 2026-10-27

**Authors:** Milenka Jean-Baptiste, H. Luz McNaughton Reyes, Tapiwa A. Tembo, Laura Nyblade, Vivian F. Go, Abigail M. Hatcher, Mike J. Chitani, Angella Mkandawire, Caroline Kumbuyo, Ireen Chirombo, Jiayu Wang, Maria H. Kim, Katie R. Mollan, Katherine R. Simon, Nora E. Rosenberg

**Affiliations:** 1 Gillings School of Global Public Health, University of North Carolina at Chapel Hill, Chapel Hill, North Carolina, United States of America; 2 Baylor College of Medicine Children’s Foundation Malawi, Lilongwe, Malawi; 3 RTI International, Research Triangle Park, Durham, North Carolina, United States of America; 4 Faculty of Health Sciences, University of the Witwatersrand, Johannesburg, South Africa; 5 Department of Pediatrics, Division of Global Health, Baylor College of Medicine, Houston, Texas, United States of America; 6 Center for AIDS Research, School of Medicine, University of North Carolina at Chapel Hill, Chapel Hill, North Carolina, United States of America; Médecins Sans Frontières (MSF), SOUTH AFRICA

## Abstract

**Background:**

Healthcare worker (HCW) HIV-related stigma can reduce participation in HIV prevention services in Malawi, including HIV index testing. However, evidence on interventions to reduce HCW stigma and improve client experiences of care in index testing settings remains limited. We evaluated HCW stigmatizing attitudes and also assessed anticipated stigma and perceptions of respectful care among both people living with HIV (index clients) and their sexual partners (contact clients) in index testing settings.

**Methods:**

We conducted a cluster randomized controlled trial across 33 clusters providing index case testing in southeastern Malawi. All HCWs received standard, in-person training. Enhanced cluster HCWs additionally received the blended learning training package consisting of tablet-based modules and in-person sessions incorporating stigma-reduction content. HCW stigmatizing attitudes were evaluated one-month (wave 1) and one-year post-training (wave 2). A subset of index and contact clients completed anticipated stigma and respectful care scales at exit interviews. Scales ranged from 0–3 (HCW stigma), 0–1 (anticipated stigma), and 1–5 (respectful care), with higher scores reflecting more stigma or respectful care. Generalized estimating equations with an identity link and gamma distribution estimated adjusted mean differences between arms.

**Results:**

We enrolled 305 HCWs, 341 index clients, and 194 contact clients. Overall stigma levels were low and respectful care ratings were high across arms. Mean HCW stigma scores were 0.8 (wave 1) and 0.7 (wave 2); adjusted mean differences between arms were −0.1 (p = 0.3) at wave 1 and 0.0 (p = 1.0) at wave 2. Index and contact client anticipated stigma means were both 0.1; adjusted mean differences between arms were 0.0 (p = 0.9) for index and 0.0 (p = 0.7) for contact clients. Respectful care means were 4.0 for index and 4.2 for contact clients; adjusted mean differences between arms were 0.0 (p = 0.9) for index and −0.1 (p = 0.3) for contact clients.

**Conclusions:**

The enhanced training package did not improve HCW or client stigma-related outcomes. Further research is needed to reduce HCW and client stigma and improve respectful care.

## Background

Despite substantial advances in HIV prevention and treatment, stigma within healthcare settings remains a critical barrier that undermines uptake of HIV services [1–4]. Malawi has made notable progress toward achieving the UNAIDS 95-95-95 targets, whereby 95% of people living with HIV know their status, 95% of these people are put on antiretroviral medications, and 95% of these people are virally suppressed by 2030 [5]. Despite gradual reductions in HIV prevalence and incidence, a gap still exists in 95% of PLWH in Malawi being aware of their status/identifying 95% the population who are living with HIV [6]. With 8.9% HIV prevalence, Malawi remains among the countries most affected by HIV in Africa and HIV and AIDS continues to be a leading cause of morbidity and mortality [6–8]. Additionally, national data indicates that approximately 25% of people living with HIV (PLWH) aged 15–49, feel ashamed of their status, alongside documented concerns about stigma and discrimination in healthcare settings [9]. Such stigma may undermine HIV service delivery by discouraging HIV status disclosure and delaying or reducing uptake of testing and treatment services [4,10–16].

These stigma related challenges are particularly relevant for HIV index case testing, a critical strategy for identifying undiagnosed HIV infections among sexual partners and biological contacts of PLWH [17,18]. In Malawi, index case testing was introduced as part of national HIV case-finding efforts and subsequently strengthened through the adoption of assisted partner notification services [19,20]. Index case testing is a focused HIV testing strategy, in which PLWH, referred to as index clients, voluntarily refer their sexual partners or family members, referred to as contact clients, for HIV testing [20]. Index case testing involves close interaction between HCWs and index clients, particularly during counseling sessions where different referral options are discussed. Referral options include assisted index case testing methods such as provider referral (HCWs reach out to contact clients directly without disclosing the index client’s identity); contract referral (index clients agree to notify contact clients themselves); and dual referral (both HCWs and index clients approach contact clients) [20]. As index case testing may involve potential disclosure of HIV status and identification of sexual partners or family members, concerns about stigma, discrimination, relationship conflict, and intimate partner violence may discourage engagement [20]. Evidence from Malawi and similar settings suggests that fear of disclosure, risk of intimate partner violence, reluctance to identify contacts, and challenges with tracing referred contacts can undermine the effectiveness of index case testing programs [21].

HIV-related stigma may influence index case testing, undermining interactions between HCWs and clients [20–24]. Stigmatizing attitudes among HCWs can increase clients’ anticipated stigma, the fear of discrimination or abuse due to one’s own stigmatized identity [25] and lower perceptions of respectful care, which centers patient rights, autonomy, dignity, and confidentiality [26–28]. When clients anticipate stigma or perceive care as disrespectful, they may be less willing to disclose information about partners and family members or engage in partner notification services due to fears of abandonment, financial loss, discrimination, or intimate partner violence [29–32], particularly if their status is inadvertently disclosed by HCWs or other family members. Contact clients may also decline testing due to similar fears or psychosocial barriers, including anxiety about a positive result or mistrust of health facilities [33]. Respectful care may, therefore, serve as an important mechanism linking HCW attitudes to client engagement in index case testing. Together, HCW stigma, client anticipated stigma, and poor perceptions of respectful care may impede effective implementation of index case testing and highlight the need for interventions that address these barriers.

Stigma-reduction training for HCWs that focuses on shifting HCW knowledge, attitudes, and beliefs about PLWH is a promising approach for improving interactions between HCWs and PLWH and shifting HCW knowledge, attitudes, and beliefs. Such trainings can improve HCW attitudes [34–40] and PLWH satisfaction with care [39], particularly when they are participatory and intentionally designed to reduce stigma. However, it remains unclear whether such trainings can reduce stigma in index case testing settings. It also remains unclear whether HCW trainings not explicitly designed to reduce stigma, but incorporating stigma-reduction elements (e.g., active listening, being non-judgmental, maintaining confidentiality, information sharing, contact with PLWH), can also impact stigma-related outcomes among HCWs and clients. Clarifying this distinction is important to inform whether future HCW trainings should be explicitly stigma-focused or whether incorporating key stigma-reduction elements into broader training initiatives is sufficient to improve client outcomes.

The PRACTICE study (Package of Resources for Assisted Contact Tracing: Implementation Costs and Effectiveness) was a cluster randomized trial evaluating the impact of a blended learning package (digital and in-person training plus continuous quality improvement) on implementation and effectiveness outcomes in Malawi’s index case testing program [41]. In comparison to the standard arm, receiving an in-person training focused on HIV testing and retention in care, PRACTICE’s blended learning training package improved HCW fidelity to index case testing implementation and strengthened index case testing outcomes, including increased testing and diagnosis of contact clients and distribution of HIV self-testing kits [42–45]. While not originally designed to reduce stigma, the blended learning training package incorporated stigma-reduction elements, such as modelling of respectful, client-centered, nonjudgemental care. This analysis has two aims to evaluate: (1) the effect of PRACTICE’s blended learning training package on HCW stigmatizing attitudes in index case testing settings, and (2) the subsequent effects on client anticipated stigma and perceptions of respectful care. In doing so, we assess the extent to which an HCW-level implementation strategy, not originally intended for stigma-reduction but having stigma-reduction elements, affects stigma outcomes.

## Methods

### Setting

The PRACTICE study was conducted in Balaka and Machinga districts in southern Malawi across Ministry of Health (MOH) and Christian Health Association of Malawi (CHAM) facilities. These facilities provide free HIV prevention, testing, care, and treatment services in line with Malawi national guidelines, including HIV testing, index case testing, and partner notification services. The facilities are supported by the Tingathe Program, a PEPFAR-funded implementing partner founded by Baylor College of Medicine Children’s Foundation Malawi, which works with the Ministry of Health to strengthen HIV service delivery [41]. Due to a critical shortage of formally trained HCWs in Malawi, HIV testing and index case testing services are largely delivered through task-shifting to lay HCWs employed by the Tingathe Program [46–48]. These lay HCWs play a central role in index case testing, including interviewing people living with HIV, eliciting and documenting contacts, providing partner notification options, and tracing contact clients through phone or community follow-up for HIV testing or linkage to care.

### Study design

Data for these analyses come from the PRACTICE study, a two-arm cluster randomized controlled trial implemented across 33 health facilities (clusters). Amongst the 33 facilities, 2 neighboring facilities were combined to form a single cluster. Thus, a total of 33 clusters were randomized in a 1:2 ratio with 11 clusters randomly assigned to the enhanced arm and 22 to the standard arm (Fig 1). Cluster level characteristics (district and facility type) were used to develop 6 strata. A 1:2 randomization occurred within each stratum to ensure a balance of cluster level characteristics between study arms. Due to the nature of the implementation strategies, blinding of participants, study staff, and investigators was not feasible [41].

**Fig 1:**
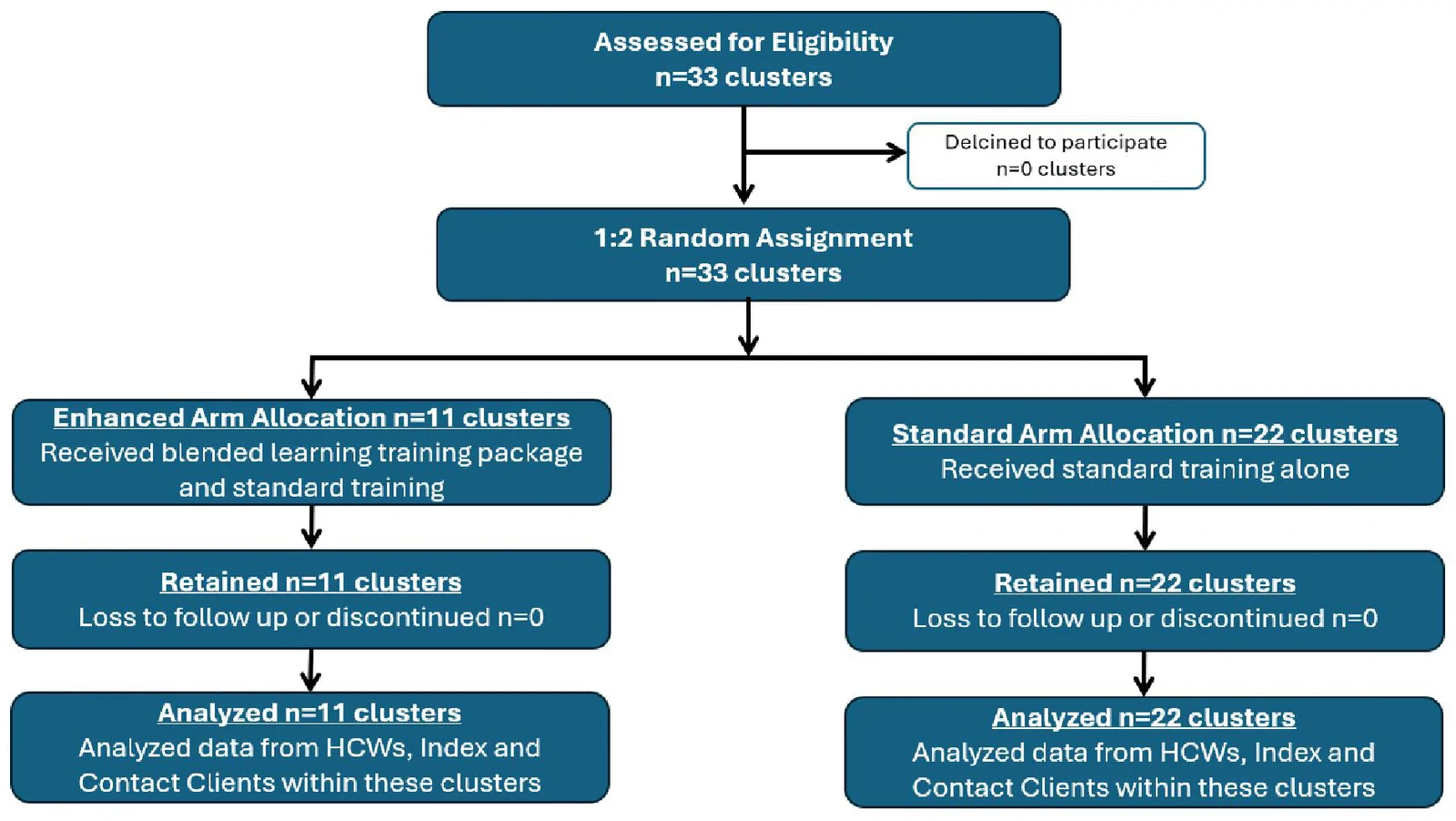
Consort diagram.

PRACTICE aimed to examine the effect of a blended learning training package on HIV index case testing implementation, service uptake, and cost-effectiveness**.** This analysis examined the effect of the blended learning training package on HCW stigma and client-level stigma and perceptions of care. Further description of the parent study is published elsewhere [41,49].

### Theory of change

This analysis was guided by the Health & Discrimination Framework [50], which posits that underlying drivers and facilitators, such as judgmental attitudes and social norms, foster stigma through labelling and communication which ultimately creates stigmatizing experiences, systems, and outcomes. We hypothesized that stigma-reduction elements embedded within PRACTICE’s blended learning training package would reduce HCW stigmatizing attitudes, thereby improving HCW–client interactions and subsequently reducing clients’ anticipated stigma and improving perceptions of respectful care (Fig 2). This hypothesis is further supported by disclosure theory, which suggests that supportive and non-judgmental healthcare interactions increase willingness to disclose HIV status and engage in care. Negative experiences may reinforce anticipated stigma and reduce disclosure to partners and family members and may also reduce perceptions of respectful care [50–54].

**Fig 2:**
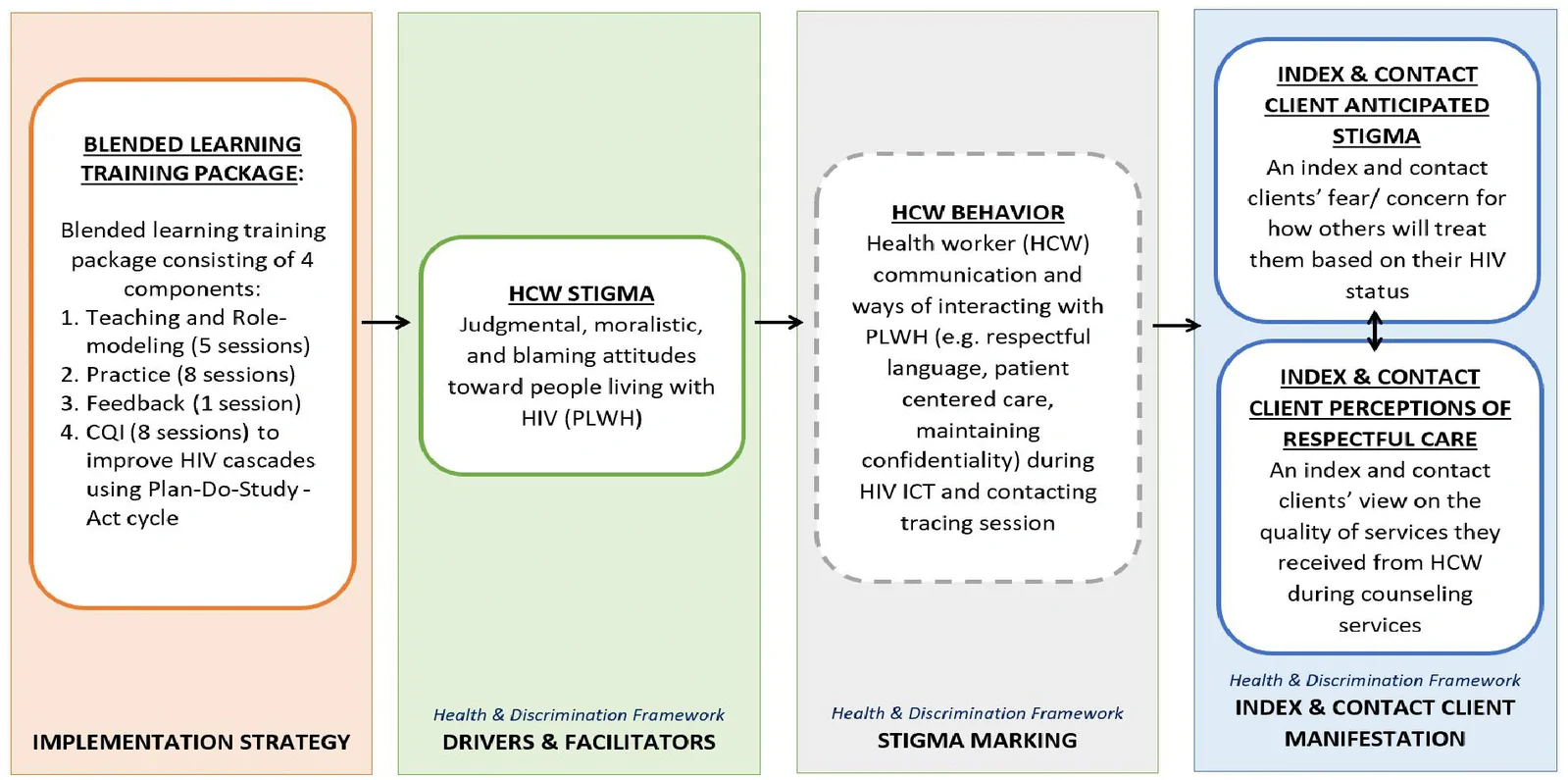
Theory of change as per health & discrimination framework.

### Training description

A year before the PRACTICE study began (October 2021), HCWs in both study arms received a 4-day centralized, in-person training which included content to create supportive relationships between the HCWs and PLWH and improve counseling skills using job aids and the development of action plans. This training included didactic content emphasizing key stigma-reduction elements, such as being non-judgmental, respecting the client, practicing active listening, creating welcoming environments, and maintaining client confidentiality.

PRACTICE’s blended learning training package was implemented at the start of the trial (July-August 2022) to enrolled HCWs in clusters randomized to the enhanced arm. The training package consisted of four components: teaching and role-modeling, practice, receiving counseling feedback from a skilled facilitator, and quality improvement. Teaching, practice, and feedback were delivered through tablet-based instruction, small-group sessions, and individualized feedback to strengthen HCWs’ index case testing capacity. Quality improvement activities engaged HCWs and facility staff in tablet-guided, in-person sessions focused on facility-level indicators. Additional details on the PRACTICE’s blended learning training package are described elsewhere [41,42,55].

Although not originally designed as a stigma-reduction intervention, the blended learning training package incorporated several components that may reduce stigma [41,42,55–57], including content emphasizing client-centered counseling, non-judgmental care, confidentiality, and supportive HCW–client interactions through instructional modules, role modeling, practice sessions, and individualized feedback. Quality improvement activities additionally engaged HCWs and facility staff in identifying and addressing facility-level barriers to index case testing implementation. Collectively, these activities reflected multiple stigma-reduction strategies described in the literature, including information provision, participatory learning, skills-building, and structural approaches [57] (S2 Table).

### Study populations

This analysis included lay HCWs and index and contact clients enrolled in the PRACTICE study. HCWs involved in delivering index case testing services were recruited from participating facilities between June and July 2022. Eligible HCWs, index clients, and contact clients were recruited consecutively from participating health facilities using standardized eligibility criteria. Consistent recruitment procedures were applied across study arms, with rolling enrollment maintained throughout the study period to include newly eligible HCWs [41].

Eligible HCWs were aged ≥18 years, worked at a study facility, and participated in Malawi’s index case testing program. Index and contact clients were recruited from ART and HIV Testing Services clinics between October 2022 and September 2023. Eligible participants were aged ≥15 years (or had guardian consent if minors), were enrolled in Malawi’s index case testing program, and provided informed consent. Contact clients included sexual partners, biological children, family members, or other social contacts referred for HIV testing by index clients. At least two index and two contact clients were enrolled per cluster, who were identified by trained research staff after liaising with facility staff through review of facility-based HTS and ART registers [41]. Participants completed an audio-recorded counseling session and an exit interview assessing anticipated stigma using the Weiser Scale and perceptions of respectful care [15,28].

### Measurement

#### Blended learning training package assessment.

Exposure to the blended learning training package was a dichotomous variable assessed through randomization assignment at the cluster level. HCWs and clients from each cluster were analyzed based on cluster assignment.

#### HCW stigmatizing attitudes assessment.

HCW stigmatizing attitudes were assessed at baseline, one-month (wave 1) and one-year post-training (wave 2) using 16-items adapted from the PopART fear and judgment stigma measure [58]. Internal consistency reliability (Cronbach’s alpha) was calculated using the current study sample. The PopART scale includes items assessing four types of stigmatizing attitudes (i.e., Feeling ashamed, perception of irresponsible behaviors, preference over to whom to provide services, and perceptions over who is deserving of services) in four distinct populations (PLWH, women who sell sex, men who have sex with men, and unmarried, young pregnant women). Response options for each item ranged from strongly disagree to strongly agree [58]. For descriptive purposes, we calculated the proportion of HCWs who “agreed” with each item in the scale (indicating endorsement of a stigmatizing belief) by coding responses of agree and strongly agree as 1 and responses of disagree and strongly disagree as 0. We also created two types of sub-scales by averaging scores across items. First, subscales for each specific stigmatizing belief (e.g., feeling ashamed) were created by averaging scores for each belief across populations. Second, subscales assessing stigmatizing attitudes for each population were created by averaging scores across attitudes for each population (e.g., PLWH). Finally, a total composite score was created by averaging responses across all 16 items (Cronbach’s alpha = 0.9).

#### Index & contact client anticipated stigma assessment.

Index and contact client anticipated stigma was assessed through the 9-item Weiser Scale [15]. The Weiser scale assesses whether PLWH felt that specific outcomes, including breakups, social exclusion, or family mistreatment, would occur if others knew of their HIV status [15,59]. The scale measures anticipated stigma from multiple sources, including intimate partners, family, and a single item for HCWs [15]. While the training package targeted HCW behavior, it was also important to assess anticipated stigma from other sources, as negative experiences with HCWs can influence how clients expect to be treated by others, including intimate partners.

Dichotomous response options for each scale item were coded as “Yes” (1) or “No” (0) [15]. A composite score was calculated averaging scores across items [59,60]. For descriptive purposes, we calculated the proportion of index or contact clients who responded “Yes” to each item in the scale (indicating endorsement of anticipated stigma). We also created a sub-scale reflecting the different sources of anticipated stigma that included intimate partners, family, and others. Anticipated stigma from HCWs were reflected in the “Other” category through one item on the scale. Scores were computed separately for index and contact clients to allow comparison across study arms. Within our analysis, a total composite score was created by averaging responses across all 9 items (Index client Cronbach’s α = 0.8, Contact client Cronbach’s α = 0.8).

#### Index & contact client perceptions of respectful care assessment.

Index and contact client perceptions of respectful care were assessed through an index case testing adapted version of the validated Perceptions of Respectful Maternity Care Scale [28]. The adapted 16-item scale consists of four sub-scales: friendly care (7-items), abuse-free care (3-items), discrimination-free care (2-items), and empowering care (4-items) [28,61]. Items in each subscale are rated on a five-point Likert scale, ranging from “Strongly Disagree” (1) to “Strongly Agree” (5). For descriptive purposes, we calculated the proportion of index or contact clients who “agreed” with each item in the scale (indicating endorsement of experiencing respectful care) by coding responses of agree and strongly agree as 1 and responses of disagree and strongly disagree as 0. Sub-scale scores for each sub-scale were also developed by summing scores across the 16 items and dividing by the number of items (16), yielding a total mean score ranging from 1 to 5, with higher scores indicating more positive perceptions of respectful care. Among contact clients, responses from the first 10 items were used to develop the total mean score since the last 6 items were for PLWH. Subscale mean scores were calculated using the same approach, averaging item responses within each defined subscale. A total composite score was created by averaging responses across all 16 items for index clients (Cronbach’s α = 0.9) and for all 10 items for contact clients (Cronbach’s α = 0.9). Cronbach’s alpha values reported in this manuscript were calculated using the current study sample.

We captured adverse events by logging their date and time, location, nature, severity, and study relatedness on study forms. Events were reported to regulatory agencies and participants were referred to appropriate services.

### Data analysis

All analyses were conducted using SAS v9.4. Analyses were conducted according to the intent-to-treat principle, with participants analyzed in their originally assigned study arm regardless of intervention exposure. We applied an α = .05 type I error rate (i.e., 95% confidence interval) with no adjustment for multiple endpoints. The stigma-related outcomes were measured as part of the larger parent PRACTICE study and were informed by a priori hypotheses. However, they were not designated as primary or key secondary confirmatory endpoints in the parent study. As such, analyses of these outcomes were not subject to formal adjustment for multiple comparisons. Item level descriptive statistics report the number and percentage of participants affirmatively responding with “Yes” to each stigma indicator in the Weiser scale and who reported “Agreement” with each item in the Perception of Respectful care scale. Less than 5% of data were missing across all outcomes. Patterns of missingness were examined and found to be non-systematic. Given the low level of missing data, analyses were conducted without imputation.

The effects of the blended learning training package on HCW stigmatizing attitudes, client anticipated stigma, and perceived respectful care were assessed by estimating adjusted mean differences in composite outcome scores between arms. Generalized estimating equations (GEE) with a gamma distribution, identity link, and exchangeable correlation structure were used to account for intra-cluster correlation at the study cluster level. For HCW stigmatizing attitudes, the model included a time-by-exposure interaction term to assess whether intervention effects differed over time. The GEE models provide adjusted mean differences with corresponding 95% confidence intervals, which serve as the primary effect estimates for continuous outcomes while accounting for clustering. This is consistent with recommendations that cluster randomized trials report effect estimates and their precision while accounting for clustering [62].

Statistical power and cluster size calculations were derived from the parent PRACTICE study and methods for determining the cluster size are described elsewhere. Detailed assumptions and parameters used in the sample size and cluster size calculations are reported in the parent PRACTICE protocol [41]. Using SAS v9.4, we calculated a minimally detectable effect size [63,64] and found the sample size for our study was enough to have 80% power to detect a difference between the enhanced and standard arms for each of the stigma outcomes in this study.

### Ethical statement

This study has been approved by the Malawi National Health Science Research Committee (#20/06/2566), University of North Carolina Institutional Review Board (20–1810) and the Baylor College of Medicine Institutional Review Board (H-48800). Voluntary, written informed consent was obtained for each participant prior to study participation. The parent study was registered at clinicaltrials.gov (NCT05343390). Additionally, de-identified data and the data dictionary are available on UNC’s Dataverse at https://doi.org/10.15139/S3/KEPG04.

## Results

### Sample characteristics

At baseline, a total of 306 lay HCWs were enrolled in the study. However, one HCW did not complete study materials. Thus, the final analytic sample included 305 HCWs, of whom 125 (41%) were in the enhanced arm and 180 (59%) were in the standard arm. Approximately half of HCWs were women (n = 159; 52%) and the median HCW age was 33.0 years (Interquartile Range (IQR): 27.0–38.0). The majority of HCWs were married (n = 219; 71.8%), identified as a community health worker/counselor (n = 237; 77.5%), had < 5 years of experience (n = 187; 61.3%), (Table 1).

**Table 1:** Baseline HCW characteristics.

	Enhanced (n = 125)	Standard (n = 180)
**Sex**		
Male	59 (47.2%)	87 (48.3%)
Female	66 (52.8%)	93 (51.7%)
**Age (years)**		
Median (IQR)	33.0 (27.0-37.0)	32.0 (26.0-39.5)
18-29	48 (38.4%)	78 (43.3%)
30-39	59 (47.2%)	57 (31.7%)
40-49	15 (12.0%)	39 (21.7%)
≥ 50	3 (2.4%)	6 (3.3%)
**Marital Status**		
Never Married/Single	31 (24.8%)	27 (15.0%)
Married	84 (67.2%)	135 (75.0%)
Divorced or separated	7 (5.6%)	13 (7.2%)
Widowed	2 (1.6%)	3 (1.7%)
No response	1 (0.8%)	2 (1.1%)
**Education Level**		
Some secondary	16 (12.8%)	27 (15.0%)
Completed Secondary	73 (58.4%)	102 (56.7%)
Any tertiary education	36 (28.8%)	51 (28.3%)
**HCW type** ^**a**^		
Community Health Worker/ Counselor	100 (80.0%)	137 (75.69%)
HIV Diagnostic Assistant	9 (7.2%)	12 (6.7%)
Site Supervisor	12 (9.6%)	22 (12.2%)
Other lay cadres (e.g.,: HTS Focal Person, Health Surveillance Assistant)	4 (3.2%)	6 (3.3%)
**Time as HCW (years)** ^**a**^		
0	16 (12.8%)	26 (14.4%)
1-2	26 (20.8%)	34 (18.9%)
3-4	41 (32.8%)	44 (24.4%)
≥ 5	42 (33.6%)	73 (40.6%)
**Time at health facility (years)** ^**a**^		
0-5	101 (80.8%)	143 (80.8%)
6-10	17 (13.6%)	30 (17.0%)
11-15	7 (5.6%)	2 (1.1%)
≥ 16	0 (0.0%)	2 (1.1%)

a Missing responses from 3 people in enhanced arm.

A total of 341 index clients were enrolled, with 124 (36.4%) in the enhanced clusters and 217 (63.6%) in the standard clusters. Overall, the median age for index clients was 34.0 years (IQR: 26.0–42.0) with 70.4% identifying as female (n = 240), and the majority being adult clients (n = 326; 95.6%). We also enrolled 194 contact clients, with 67 (34.5%) in the enhanced clusters and 127 (65.5%) in the standard clusters. Over half of contact clients were male (n = 108, 55.7%) and 162 were adults (83.5%). Median age for all adult contact clients was 37.0 years (IQR: 28.0–47.0). Approximately 16.5% of contact clients (n = 32) were parents/guardians of pediatric contact clients who did not report their children’s age (Table 2).

**Table 2:** Index and contact client demographic characteristics.

n (%) throughout	Index Clients (n = 341)		Contact Clients (n = 194)	
	Enhanced (n = 124)	Standard (n = 217)	Enhanced (n = 67)	Standard (n = 127)
**Entry Point** ^b^				
HIV Testing Services (HTS)	69 (55.7%)	113 (52.1%)		
Antiretroviral Treatment (ART)	55 (44.4%)	104 (47.9%)		
Community Setting			25 (37.3%)	44 (34.7%)
Clinic Setting			42 (62.7%)	83 (65.4%)
**Client Type**				
Adult client	119 (96.0%)	207 (95.4%)	58 (86.6%)	104 (81.9%)
Pediatric client	5 (4.0%)	10 (4.6%)	9 (13.4%)	23 (18.1%)
**Sex**				
Male	43 (34.7%)	58 (26.7%)	42 (62.7%)	66 (52.0%)
Female	81 (65.3%)	159 (73.3%)	25 (37.3%)	61 (48.0%)
**Age of client/guardian (years)** ^**c**^				
Median Age (Q1; Q3)	35.0 (27.0; 45.0)	33.0 (26.0; 40.0)	39.0 (29.0; 48.0)	35.0 (27.5; 46.5)
0-14	5 (4.0%)	10 (4.6%)	9 (13.4%)	23 (18.1%)
15-29	36 (29.0%)	69 (31.8%)	15 (22.4%)	29 (22.8%)
30-39	37 (29.8%)	77 (35.5%)	15 (22.4%)	31 (24.4%)
40-49	26 (21.0%)	34 (15.7%)	15 (22.4%)	25 (19.7%)
≥ 50	20 (16.1%)	27 (12.4%)	13 (19.4%)	19 (15.0%)
**Marital Status**				
Never Married/Single	5 (4.0%)	9 (4.2%)	1 (1.5%)	7 (5.5%)
Married	73 (58.9)	131 (60.4%)	52 (77.6%)	87 (68.5%)
Divorced or separated	32 (25.8%)	55 (25.4%)	4 (6.0%)	9 (7.1%)
Widowed	9 (7.3%)	12 (5.5%)	1 (1.5%)	1 (0.8%)
Child	5 (4.0%)	10 (4.6%)	9 (13.4%)	23 (18.1%)
**Education Level** ^**c, d**^				
No School	24 (19.4%)	43 (19.8%)	6 (10.3%)	19 (18.1%)
Some primary school	66 (53.2%)	129 (59.5%)	32 (55.1%)	64 (61.5%)
Completed primary school	8 (6.5%)	13 (6.0%)	9 (15.5%)	6 (5.7%)
Any secondary or tertiary education	21 (16.9%)	22 (10.1%)	11 (19.0%)	15 (14.3%)
**Client Most Recent HIV Test** ^**e**^				
Day of survey	68 (54.8%)	113 (52.1%)	66 (98.5%)	127 (100.0%)
< 1 year ago	5 (4.0%)	7 (3.3%)	1 (1.5%)	0 (0.0%)
≥ 1 year ago	50 (40.3%)	97 (44.7%)	0 (0.0%)	0 (0.0%)
**Client Most Recent HIV Test Result** ^**e**^				
Negative	0 (0.0%)	0 (0.0%)	63 (94.0%)	112 (88.1%)
Positive	123 (99.2%)	216 (99.5%)	4 (6.0%)	13 (10.3%)
Indeterminate	0 (0.0%)	1 (0.5%)	0 (0.0%)	2 (1.6%)

a Missing responses from 14 clients (8 Enhanced index clients and 6 Enhanced contact clients).

b Greyed cells = Not Applicable.

c Missing responses from 32 contact clients (9 in Enhanced arm and 23 in Standard arm) reflecting child contact client.

d Missing responses from 15 child index clients (5 in Enhanced arm and 10 in Standard arm) whose parent/guardians completed the form on their behalf).

e Missing 1 response from index client.

f Missing 6 responses from the Contact enhanced arm.

### HCW stigmatizing attitudes

Across both study arms and all timepoints, fewer than half of HCWs endorsed any of the 16 individual stigmatizing statements, with agreement ranging from 3.4% to 43.2% (S3 Table). Across all three timepoints, in both study arms, the most frequently endorsed items were the attitudes women who sell sex and men who have sex with men behave irresponsibly. For women who sell sex, endorsement of the belief that this behavior is irresponsible was 29.1% at baseline, 25.6% at Wave 1, and 28.0% at Wave 2. For men who have sex with men, endorsement of the belief that this behavior is irresponsible was 41.1% at baseline 30.1% at Wave 1 and 28.0% at Wave 2. Additionally, across all time points, a large percentage of HCWs reported they would feel ashamed if a family member were a woman who sold sex (Baseline: 29.1%; Wave 1: 19.0%; Wave 2: 20.3%), or a man who has sex with men (Baseline: 36.1%; Wave 1: 22.5%; Wave 2: 25.5%).

Contrary to expectations, composite HCW stigma scores did not differ significantly by study arm at any time point (Baseline Adjusted Mean Difference: −0.1, 95% CI: −0.2, 0.1; p = 0.42; Wave 1 Adjusted Mean Difference: −0.1, 95% CI: −0.2, 0.1; p = 0.28; Wave 2 Adjusted Mean Difference: −0.0, 95% CI: −0.1, 0.1; p = 0.97) (Table 3). This pattern was consistent across all population groups assessed (PLWH, women who sell sex, men who have sex with men, and unmarried pregnant young women) and across stigmatizing attitudes (shame, perceptions of irresponsibility, beliefs about service deservingness, and reluctance to provide care).

**Table 3:** Effect of blended learning training package on HCW stigmatizing attitudes.

	Wave 0				Wave 1				Wave 2			
	Enhanced Mean (95% CI)	Standard Mean (95% CI)	Adj Mean Difference (95% CI)	p-value	Enhanced Mean (95% CI)	Standard Mean (95% CI)	Adj Mean Difference (95% CI)	p-value	Enhanced Mean (95% CI)	Standard Mean (95% CI)	Adj Mean Difference (95% CI)	p-value
***HCW STIGMATIZING ATTITUDES TOWARDS…***												
People Living with HIV (range: 0–3)	0.7 (0.6, 0.8)	0.7 (0.6, 0.7)	0.0 (−0.1, 0.1)	0.93	0.6 (0.5, 0.7)	0.6 (0.5, 0.7)	−0.1 (−0.2, 0.1)	0.44	0.6 (0.5, 0.7)	0.5 (0.4, 0.6)	−0.1 (−0.2, 0.1)	0.37
Women who sell sex (range: 0–3)	1.0 (0.9, 1.1)	1.0 (0.9, 1.0)	−0.1 (−0.2, 0.1)	0.32	0.8 (0.7, 0.9)	0.8 (0.7, 0.9)	0.0 (−0.2, 0.1)	0.66	0.8 (0.7, 0.9)	0.8 (0.7, 0.8)	0.0 (−0.2, 0.1)	0.65
Men who have sex with Men (range: 0–3)	1.0 (0.9-1.2)	1.0 (0.9-1.1)	0.0 (−0.2, 0.1)	0.85	0.9 (0.8, 1.0)	0.8 (0.4, 0.8)	−0.1 (−0.2, 0.1)	0.28	0.8 (0.7-0.9)	0.9 (0.8, 1.0)	0.1 (−0.1, 0.2)	0.22
Young Women who are pregnant before marriage (range: 0–3)	1.0 (0.9, 1.1)	0.9 (0.8, 1.0)	−0.1 (−0.2, 0.0)	0.12	0.8 (0.7, 1.0)	0.7 (0.7, 0.8)	−0.1 (−0.2, 0.0)	0.14	0.7 (0.6, 0.9)	0.7 (0.7, 0.8)	0.0 (−0.1, 0.1)	0.88
***SCALE DIMENSION***												
I would be **ashamed** if someone in my family was a… (0–3)	1.0 (0.8, 1.1)	0.9 (0.9, 1.0)	0.0 (−0.2, 0.1)	0.84	0.9 (0.7, 1.0)	0.8 (0.7, 0.9)	−0.1 (−0.2, 0.1)	0.25	0.8 (0.7, 0.9)	0.8 (0.7, 0.9)	0.0 (−0.2, 0.1)	0.89
**…irresponsible** behaviors (0- 3)	1.2 (1.0, 1.3)	1.0 (0.9, 1.1)	−0.1 (−0.2, 0.0)	0.08	1.0 (0.8, 1.1)	0.9 (0.8, 1.0)	−0.1 (−0.2, 0.1)	0.22	0.9 (0.7, 1.0)	0.9 (0.7, 1.0)	0.0 (−0.1, 0.2)	0.69
Other people **deserve** access to health services more than … (0–3)	1.0 (0.9, 1.2)	1.0 (0.9, 1.1)	0.0 (−0.2, 0.2)	0.84	0.9 (0.8, 1.1)	0.8 (0.7, 0.9)	−0.1 (−0.3, 0.1)	0.34	0.8 (0.7, 0.9)	0.8 (0.7, 0.9)	0.0 (−0.2, 0.1)	0.74
If I had a choice, I would **prefer not to provide services** to … (0–3)	0.5 (0.4, 0.6)	0.5 (0.4, 0.6)	0.0 (−0.2, 0.1)	0.62	0.5 (0.7, 0.9)	0.5 (0.4, 0.6)	0.0 (−0.1, 0.1)	0.92	0.5 (0.4, 0.6)	0.5 (0.4, 0.6)	0.0 (−0.1, 0.1)	0.98
**TOTAL MEAN SCORE** **(RANGE: 0–3)**	**0.9** **(0.8, 1.0)**	**0.9** **(0.8, 0.9)**	**−0.1** **(−0.2, 0.1)**	**0.42**	**0.8** **(0.7, 0.9)**	**0.7** **(0.7, 0.8)**	**−0.1** **(−0.2, 0.1)**	**0.28**	**0.7** **(0.6, 0.8)**	**0.7** **(0.7, 0.8)**	**0.0** **(−0.1, 0.1)**	**0.97**

### Index & contact client anticipated stigma & perceptions of respectful care

#### Anticipated stigma.

Across both study arms, less than one-third of index and contact clients reported anticipated stigma, with agreement on any given item ranging from 3.7% to 28.4% (S4 Table). In both study arms, the most frequently endorsed item was fear of a partner breakup, reported by 22.4% of index clients and 27.8% of contact clients. Fear of being treated badly by HCWs because of HIV disclosure was reported by a smaller percentage of clients (5.3% of index clients and 9.3% of contact clients). Client endorsements of other items (i.e.,: being treated badly by HCWs, work, or school, losing your job or friends, or being a social outcast) ranged from 6%−16% amongst index clients and 4%−8% amongst contact clients. Of the three sources of anticipated stigma (partner, family, and community), clients across both arms reported fear of negative reactions from one’s partner most frequently (25.9% of index clients; 30.4% of contact clients). Across both study arms, 36.1% of index clients and 42.8% of contacted clients responded with “Yes” to at least one item in the scale, reflecting some level of anticipated stigma.

Contrary to expectations, there was no difference in average anticipated stigma scores between the enhanced and standard arms for either index or contact clients (Index Client Adjusted Mean Difference: 0.0, 95% CI: −0.1, 0.1; p = 0.91; Contact client Adjusted Mean Difference: 0.0; 95% CI: −0.1, 0.1, p = 0.66; Table 4).

**Table 4:** Effect of blended learning training package on client anticipated stigma & perceptions of respectful care.

	Index Clients (n = 340)				Contact Clients (n = 194)			
	Enhanced Mean (95% CI)	Standard Mean (95% CI)	Adj Mean Difference (95% CI)	p-value	Enhanced Mean (95% CI)	Standard Mean (95% CI)	Adj Mean Difference (95% CI)	p-value
**ANTICIPATED STIGMA SUB-SCALES** *Anticipated any stigma from…*								
Partner mean (items 6 and/or 7) (range: 0–1)	0.2 (0.1, 0.2)	0.2 (0.2, 0.3)	0.0 (−0.2, 0.1)	0.50	0.2 (0.1, 0.3)	0.2 (0.2, 0.3)	0.0 (−0.1, 0.1)	0.83
Family mean (items 3 and/or 4) (0-1)	0.1 (0.0, 0.1)	0.1 (0.1, 0.1)	−0.1 (−0.2, 0.1)	0.36	0.1 (0.0, 0.2)	0.1 (0.0, 0.1)	0.0 (−0.1, 0.1)	0.48
Others mean (items 1,2,5,8, and/or 9) (0-1)	0.1 (0.1, 0.2)	0.1 (0.1, 0.1)	0.2 (−0.1, 0.2)	0.65	0.1 (0.1, 0.2)	0.1 (−0.1, 0.1)	0.0 (−0.1, 0.1)	0.51
**ANTICIPATED STIGMA** **TOTAL MEAN SCORE (0–1)**	**0.1** **(0.1, 0.2)**	**0.1** **(0.1, 0.2)**	**0.0** **(−0.1, 0.1)**	**0.91**	**0.1** **(0.1, 0.2)**	**0.1** **(0.1, 0.2)**	**0.0** **(−0.1, 0.1)**	**0.66**
**PERCEPTIONS OF RESPECTFUL CARE SUB-SCALES**								
Friendly Care mean (items 1–7) (range: 1–5)	4.1 (4.0, 4.1)	4.1 (4.0, 4.2)	0.0 (−0.1, 0.1)	0.72	4.1 (4.0, 4.2)	4.2 (4.1, 4.2)	−0.1 (−0.2, 0.1)	0.50
Abuse Free Care mean (items 8–10) (1-5)	4.1 (4.0, 4.2)	4.1 (4.1, 4.2)	0.0 (−0.1, 0.1)	0.50	4.1 (4.0, 4.2)	4.2 (4.1, 4.3)	−0.1 (−0.2,0.1)	0.23
Discrimination Free Care mean (items 11–12) (1-5)	4.0 (4.0, 4.1)	4.1 (4.0, 4.1)	0.0 (−0.1, 0.1)	0.51				
Empowering Care mean (items 13–15) (1-5)	3.9 (3.8, 4.0)	3.9 (3.8, 4.0)	0.1 (−0.1, 0.3)	0.50				
**PERCEPTIONS OF RESPECTFUL CARE** **TOTAL MEAN SCORE (1–5)**	**4.0** **(4.0, 4.1)**	**4.0** **(4.0, 4.1)**	**−0.0** **(−0.1, 0.1)**	**0.93**	**4.1** **(4.0, 4.2)**	**4.2** **(4.1, 4.3)**	**−0.1** **(−0.2, 0.1)**	**0.29**

#### Perceptions of respectful care.

Perceptions of respectful care amongst index and contact clients across both arms ranged from 56.5–100.0% of client endorsement for any individual item (S5 Table). Across both study arms, all of index and contact clients responding to this survey, felt that their HCW treated them with respect as individuals. Nearly all index and contact clients in both arms felt that their HCW treated them with a kind approach, in a friendly manner, and spoke to them in a language they could understand.

Findings suggest there were no differences in perceptions of respectful care between the enhanced and standard arms for either index or contact clients (Index client Adjusted mean difference: 0.0, 95%CI: −0.1, 0.1, p = 0.93; Contact client Adjusted mean difference: −0.1, 95%CI: −0.2, 0.1, p = 0.29; Table 4). This pattern of findings held across sub-scales.

## Discussion

Our study examined how a blended learning training package, not explicitly designed to reduce stigma but incorporating key stigma-reduction elements, influenced HCW stigmatizing attitudes in index case testing settings. We also examined whether the blended learning training package indirectly influenced clients’ general anticipated stigma and perceptions of respectful care. Contrary to expectations, the PRACTICE study’s blended learning training package had no evident impacts on HCW stigmatizing attitudes, nor on index and contact client anticipated stigma or perceptions of respectful care. Across all three timepoints, HCWs in both study arms consistently reported relatively low levels of stigmatizing attitudes. Similarly, both index and contact clients reported low anticipated stigma and moderate to high levels of perceived respectful care. Despite overall low stigma scores, findings revealed persistent HCW judgment, particularly toward PLWH with intersecting, marginalized identities.

Prior studies of blended learning interventions have demonstrated improvements in HCW knowledge acquisition [65] and reductions in stigma [36,66], particularly in settings with higher baseline stigma levels. In contrast, HCWs in our study consistently reported low stigmatizing attitudes across all timepoints. Although quantitative data on HCW stigma in Malawi are lacking, qualitative studies have documented HCW stigma and bias in this context [67,68], providing contextual support for our results. Similarly, the low levels of client anticipated stigma observed in our study align with prior research in Blantyre, Malawi, which reported low to moderate anticipated stigma among PLWH [69]. These low anticipated stigma levels may reflect increased access to HIV counseling and testing, expanded availability of antiretroviral therapy, and greater community knowledge of HIV [69], which may reduce expectations of overt stigma or judgment. Despite overall low stigma levels, we observed slightly higher HCW stigma toward PLWH with intersecting, marginalized identities, underscoring the persistence of more nuanced or context-specific forms of judgment. Together, these findings suggest that blended learning approaches not explicitly designed to address intersectional stigma may be insufficient to shift more deeply embedded or context-specific biases. They also highlight how even in low-stigma contexts, targeted interventions may be needed to prevent the emergence of stigma and to sustain positive attitudes over time.

In the larger PRACTICE trial we observed substantial behavior change with the blended learning training package. Specifically, HCWs in the enhanced arm were considerably more compliant with the index case testing protocols than those in the standard arm [42]. Additionally, as compared to HCWs in the standard arm, HCWs in the enhanced arm had more contact clients elicited and tested and distributed more HIV self-test kits [43]. These findings suggest that the blended learning training package improved HCW ability to implement index case testing protocols [44,45].

Despite these positive effects, we observed no significant differences in HCW stigmatizing attitudes between arms. There are several potential explanations for this finding. First, the low stigma levels observed may have limited our ability to see further declines in stigma. Systematic reviews of stigma-reduction interventions report that many interventions yield small-to-moderate effects, and these modest effects are more difficult to detect when stigma is low, measurement tools have limited sensitivity, or populations already show relatively favorable baseline attitudes [70,71]. The training was also lacking in some stigma-reduction elements, such as engaging PLWH in the implementation of the training, or empowerment approaches used to improve client coping mechanisms for overcoming stigma which are proven stigma-reduction methods [36,56,72]. Additionally, although the blended-learning training package enhanced general interpersonal skills (e.g., being non-coercive and non-judgmental), it did not focus on challenging existing perceptions and biases towards PLWH [73–75]. HCW-level stigma-reduction may also require intentionally focusing on stigma-reduction from the outset of intervention design. Although PRACTICE’s blended learning training package included stigma-reduction elements conveyed and practiced in its modules, stigma-reduction was not the original intention of the package. Several rigorously tested, stigma-reduction interventions which have successfully reduced HCW stigma have intentionally designed the intervention for HCW stigma-reduction [34–40,72].

Client-level interventions may be needed to directly address client-level anticipated stigma, rather than indirectly doing so through HCWs, as done in this study. Two studies, the Collaborative HIV prevention and Adolescent Mental Health Family Program (CHAMP) study [76,77] and the Sustainable East Africa Research in Community Health (SEARCH) study [77], successfully reduced anticipated stigma around HIV disclosure in 3 countries in East and Southern Africa. CHAMP administered an 8-session, interactive training called “Vuka” to youth in a community setting in South Africa, resulting in a greater decrease in anticipated stigma in the intervention group. SEARCH, conducted in Kenya and Uganda, expanded community-based HIV testing and found that universal testing normalized HIV testing, increased self-disclosure among PLWH due to enhanced social support, and may have mitigated anticipated stigma. Both studies suggest that focusing directly on client-level interventions and incorporating community support can help reduce anticipated stigma in HIV testing contexts [76,77].

This analysis also allows us to understand stigma in the index case testing space in Malawi in several ways. To our knowledge, this is the only study systematically assessing the impact of an intervention on stigma in the index case testing setting. Particularly, to our knowledge no studies have assessed the impact of a HCW training on both HCWs and PLWH stigma in index case testing settings. Additionally, while multiple studies have assessed anticipated stigma for HIV outcomes in Malawi [68,69], none have rigorously and quantitatively assessed the effect of an intervention on stigma levels.

### Strengths & limitations

A study strength is its cluster randomized controlled trial design which enabled rigorous evaluation of the blended learning training package and contribute to the limited body of RCT evidence on interventions to reduce HCW stigma in Africa [78–80]. Additional strengths include the use of psychometrically validated measures to assess HCW stigmatizing attitudes, client anticipated stigma, and perceptions of respectful care [28,58,60,81]. Although the study used established measures, some instruments were adapted to enhance contextual relevance for the study setting; consequently, the psychometric properties of the adapted versions may not fully reflect those of the original validated scales. Several additional limitations should be considered. The Weiser Scale included only one item specifically assessing anticipated stigma from HCWs, limiting our ability to isolate the influence of HCW interactions on client anticipated stigma. To address this, we complemented the measure with the Perceptions of Respectful Care Scale to better capture clients’ experiences with providers. In addition, both HCW and client responses may have been influenced by social desirability bias, particularly given the patient-centered focus of the intervention and the sensitivity of stigma-related questions [82–84]. Although HCW surveys were self-administered and the Weiser Scale was designed to minimize response bias [15,59], participants may still have underreported stigmatizing attitudes or negative care experiences.

## Conclusions

The blended learning training package did not substantially effect HCW stigmatizing attitudes or client-reported anticipated stigma and perceptions of respectful care in index case testing. These findings should be interpreted in the context of generally low baseline stigma levels among both HCWs and clients, which may have limited the ability to detect further reductions and may reflect prior exposure to stigma-reduction efforts. The results suggest that embedding limited stigma-related content within broader training interventions may be insufficient to produce measurable change, and that more targeted stigma-reduction approaches, including meaningful engagement with people living with HIV, may be required. Despite the null findings, this study addresses an important gap in the limited literature on stigma reduction in the index case testing context and highlights the importance of assessing intersectional stigma, even when stigma levels are low. Given the role of stigma as a barrier to index case testing uptake and HIV service engagement, future research should evaluate intentionally designed, multi-level stigma-reduction strategies to improve participation in index case testing and reduce stigma effectively.

## Supporting information

S1 File CONSORT checklist. (DOCX)

S2 Table PRACTICE training package. (DOCX)

S3 Table HCW descriptive table. (DOCX)

S4 Table PLWH anticipated stigma descriptive table. (DOCX)

S5 Table PLWH respectful care descriptive table. (DOCX)
