# Supplementary material for: Assessing stigma and perceptions of respectful care in Malawi’s index case testing program: Findings from a cluster randomized controlled trial
Source: PLoS One. 2026 Oct 27;21(10):e0345705. doi: 10.1371/journal.pone.0345705 (PMC13712766; doi:10.1371/journal.pone.0345705)
Supplement: Supplementary file 2 [file pone.0345705.s002.docx]

## S2 Supplementary File 2. PRACTICE Blended Learning Training Package Components & and Their Potential Influence on HCW Stigmatizing Attitudes

| **Training Components** | **Component Goal(s)** | **Related stigma-reduction messages and concepts conveyed during component** | **Stigma-reduction Element**  **Employed in Session** [55] |
| --- | --- | --- | --- |
| 1. Teaching & Role Modelling | Impart knowledge of index case testing skills, protocols, and professional behaviors using culturally embedded scenarios and demonstrating desired behaviors. | - Active listening - Being non-judgmental - Being client centered - Being non-coercive - Treating clients with dignity and respect - Different types of populations deserve HIV testing and care - Maintaining client confidentiality - Building rapport with client and normalizing discussions with them - Making client feel welcomed, safe, supported, and empowered - Conveying respect and building trust - Ensuring counseling session is voluntary - Being respectful of boundaries - Being culturally and linguistically appropriate - Having a cohesive communication style and flow | 🗹 Provision of information  🗹 Participatory learning  🗹 Skills-building activities  🞎 Contact w/ stigmatized group  🞎 Empowerment approach  🞎 Structural” or “policy change |
| 2. Practice | Apply and implement index case testing knowledge gained through case-based learning |  |  |
| 3. Counselling Feedback | To identify challenges shortcomings and provide feedback on areas in need of improvement for high quality counselling |  |  |
| 4. Quality Improvement | To identify structural/ facility level indicators to address index case testing issues through action plans | - Stigma-related content was not formally integrated into these sessions, but it was expected to come up in discussions of cascade barriers that could potentially influence clinic-level policies | 🗹 Provision of information  🗹 Participatory learning  🗹 Skills-building activities  🞎 Contact w/ stigmatized group  🞎 Empowerment approach  🗹 Structural” or “policy change |
