# Supplementary material for: Assessing stigma and perceptions of respectful care in Malawi’s index case testing program: Findings from a cluster randomized controlled trial
Source: PLoS One. 2026 Oct 27;21(10):e0345705. doi: 10.1371/journal.pone.0345705 (PMC13712766; doi:10.1371/journal.pone.0345705)
Supplement: Supplementary file 3 [file pone.0345705.s003.docx]

S3 Supplementary File 3. HCW Stigmatizing Attitudes Frequencies & Effect of Blended Learning Training Package on HCW Stigmatizing Attitudes

| **Dichotomous PopART Item**  **Agreed, n (%)** | **Baseline** | | **Wave 1** | | **Wave 2** | |
| --- | --- | --- | --- | --- | --- | --- |
|  | **Enhanced**  **(n=125)** | **Standard**  **(n=177)^a^** | **Enhanced**  **(n=117)^b^** | **Standard**  **(n=172)^c^** | **Enhanced**  **(n=115)^d^** | **Standard**  **(n=171)^e^** |
| E1. I would be ashamed if someone in my family was a person living with HIV (PLHIV). | 9 (7.2%) | 11 (6.2%) | 13 (11.1%) | 10 (5.8%) | 11 (9.6%) | 6 (3.5%) |
| E2. PLHIV engage in irresponsible behaviors. | 19 (15.2%) | 19 (10.7%) | 11 (9.4%) | 17 (9.9%) | 10 (8.7%) | 12 (7.0%) |
| E3. Other people deserve access to health services more than PLHIV. | 37 (29.6%) | 53 (29.9%) | 34 (29.1%) | 39 (22.7%) | 19 (16.5%) | 31 (18.1%) |
| E4. If I had a choice, I would prefer not to provide services to PLHIV. | 6 (4.8%) | 6 (3.4%) | 7 (6.0%) | 5 (2.9%) | 7 (6.1%) | 3 (1.8%) |
| E5. I would be ashamed if someone in my family was a woman who sold sex. | 36 (28.8%) | 52 (29.4%) | 23 (19.7%) | 32 (18.6%) | 27 (23.5%) | 31 (18.1%) |
| E6. Women who sell sex engage in irresponsible behaviors. | 54 (43.2%) | 55 (31.1%) | 29 (24.8%) | 45 (26.2%) | 30 (26.1%) | 50 (29.2%) |
| E7. Other people deserve access to health services more than women who sell sex. | 41 (32.8%) | 52 (29.4%) | 23 (19.7%) | 29 (16.9%) | 19 (16.5%) | 20 (11.7%) |
| E8. If I had a choice, I would prefer not to provide services to women who sell sex. | 7 (5.6%) | 5 (2.8%) | 5 (4.3%) | 5 (2.9%) | 6 (5.2%) | 1 (0.6%) |
| E9. I would be ashamed if someone in my family was a man who has sex with men. | 42 (33.6%) | 67 (37.9%) | 29 (24.8%) | 36 (20.9%) | 24 (20.9%) | 49 (28.7%) |
| E10. Men who have sex with men engage in irresponsible behaviors. | 54 (43.2%) | 70 (39.6%) | 38 (32.5%) | 49 (28.5%) | 26 (22.6%) | 54 (31.6%) |
| E11. Other people deserve access to health services more than men who have sex with men. | 35 (28.0%) | 56 (31.6%) | 27 (23.1%) | 29 (16.9%) | 18 (15.7%) | 28 (16.4%) |
| E12. If I had a choice, I would prefer not to provide services to men who have sex with men. | 8 (6.4%) | 9 (5.1%) | 5 (4.3%) | 7 (4.1%) | 4 (3.5%) | 10 (5.9%) |
| E13. I would be ashamed if someone in my family was a young woman who became pregnant before marriage. | 36 (28.8%) | 40 (22.6%) | 26 (22.2%) | 24 (14.0%) | 17 (14.8%) | 23 (13.5%) |
| E14. Young women who become pregnant before marriage engage in irresponsible behaviors. | 37 (29.6%) | 40 (22.6%) | 26 (22.2%) | 26 (15.1%) | 16 (13.9%) | 25 (14.6%) |
| E15. Other people deserve access to health services more than young women who become pregnant before marriage. | 37 (29.6%) | 48 (27.1%) | 24 (20.5%) | 32 (18.6%) | 13 (11.3%) | 25 (14.6%) |
| E16. If I had a choice, I would prefer not to provide services to young women who become pregnant before marriage. | 4 (3.2%) | 9 (5.1%) | 4 (3.4%) | 7 (4.1%) | 3 (2.6%) | 5 (2.9%) |

a missing 3 participants in the standard arm at baseline	b missing 8 from enhanced at wave 1	c missing 8 from standard at wave 1

d missing 10 from enhanced at wave 2	e missing 9 from standard at wave 2
