# Supplementary material for: Assessing stigma and perceptions of respectful care in Malawi’s index case testing program: Findings from a cluster randomized controlled trial
Source: PLoS One. 2026 Oct 27;21(10):e0345705. doi: 10.1371/journal.pone.0345705 (PMC13712766; doi:10.1371/journal.pone.0345705)
Supplement: Supplementary file 4 [file pone.0345705.s004.docx]

S4 Supplementary File 4. Client Anticipated Stigma Frequencies & Means

| **9-item Weiser Scale prompt:** Do you think any of the following things might happen to you if others found out about your HIV status? Do you think you would: | **Index Clients (n=340)** | | **Contact Clients (n=194)** | |
| --- | --- | --- | --- | --- |
|  | **Enhanced Yes n (%)** | **Standard Yes n (%)** | **Enhanced Yes n (%)** | **Standard Yes n (%)** |
| *Items* |  |  |  |  |
| 1. Be treated badly by health workers (O)^a,b^ | 10 (8.1%) | 8 (3.7%) | 4 (6.0%) | 14 (11.0%) |
| 2. Lose your job or livelihood (O)^a,b^ | 6 (4.9%) | 16 (7.4%) | 7 (10.5%) | 8 (6.3%) |
| 3. Be denied care by family if sick (F)^a,b^ | 8 (6.5%) | 17 (7.8%) | 5 (7.5%) | 8 (6.3%) |
| 4. Be rejected by family (F)^a,b^ | 12 (9.8%) | 21 (9.7%) | 7 (10.5%) | 9 (7.1%) |
| 5. Be treated badly at work or school (O)^a,b^ | 13 (10.6%) | 28 (12.9%) | 11 (16.4%) | 15 (11.8%) |
| 6. Be physically abused by your partner (P)^a,b^ | 14 (11.4%) | 34 (15.7%) | 8 (11.9%) | 16 (12.6%) |
| 7. Experience break-up of your relationship (P)^a,b^ | 24 (19.5%) | 52 (24.0%) | 18 (26.9%) | 36 (28.4%) |
| 8. Become a social outcast (O)^a,b^ | 22 (17.9%) | 31 (14.3%) | 10 (14.9%) | 15 (11.8%) |
| 9. Lose your friends (O)^a,b^ | 17 (13.8%) | 24 (11.1%) | 11 (16.4%) | 14 (11.0%) |
| Answered “Yes” to one or more of the above | 43 (34.7%) | 80 (36.9%) | 28 (41.8%) | 55 (43.3%) |

a 1 missing from enhanced arm from index clients

b (O)=Other, (F)=Family, (P)=Partner
