# Supplementary material for: Assessing stigma and perceptions of respectful care in Malawi’s index case testing program: Findings from a cluster randomized controlled trial
Source: PLoS One. 2026 Oct 27;21(10):e0345705. doi: 10.1371/journal.pone.0345705 (PMC13712766; doi:10.1371/journal.pone.0345705)
Supplement: Supplementary file 5 [file pone.0345705.s005.docx]

S5 Supplementary File 5. Client Perceptions of Respectful Care Item Frequencies & Means

|  | **Index Clients (n=341)** | | **Contact Clients (n=194)** | |
| --- | --- | --- | --- | --- |
|  | **Enhanced**  **n/124 (%)**  **Agree** | **Standard**  **n/217 (%)**  **Agree** | **Enhanced**  **n/67 (%)**  **Agree** | **Standard**  **n/127 (%)**  **Agree** |
| ***Friendly Care*** |  |  |  |  |
| 1. I felt that health worker treated me with a kind approach.^a^ | 124 (100.0%) | 216 (100.0%) | 67 (100.0%) | 127 (100.0%) |
| 2. The health worker treated me in a friendly manner.^a^ | 124 (100.0%) | 216 (100.0%) | 67 (100.0%) | 127 (100.0%) |
| 3. The health worker talked positively about my HIV status.^b,e^ | 113 (95.0%) | 204 (99.0%) | 4 (100.0%) | 11 (100.0%) |
| 4. The health worker showed his/her concern and empathy. | 123 (99.2%) | 209 (96.3%) | 64 (95.6%) | 127 (100.0%) |
| 5. The health worker treated me with respect as an individual. | 124 (100.0%) | 217 (100.0%) | 67 (100.0%) | 127 (100.0%) |
| 6. The health workers spoke to me in a language that I could understand. | 124 (100.0%) | 214 (98.6%) | 67 (100.0%) | 127 (100.0%) |
| 7. The health worker called me by my name. | 76 (61.3%) | 123 (56.7%) | 34 (50.8%) | 70 (56.5%) |
| ***Abuse Free Care*** |  |  |  |  |
| 8. The health worker explained that my privacy would be protected. ^c^ | 121 (98.4%) | 215 (99.1%) | 62 (92.5%) | 125 (98.5%) |
| 9. The health workers threatened me if I did not obey. % DISAGREEING (R) ^c,f^ | 122 (99.2%) | 212 (97.7%) | 67 (100.0%) | 126 (98.5%) |
| 10. The health workers shouted at me because of my behaviors. % DISAGREEING (R) ^c,f^ | 122 (99.2%) | 211 (97.2%) | 67 (100.0%) | 125 (99.2%) |
| ***Discrimination Free Care*** |  |  |  |  |
| 11. Some of the health workers did not treat me well because of my HIV status. % DISAGREEING (R) ^d,e,f^ | 117 (99.2%) | 201 (97.1%) | Not applicable to contacts | Not applicable to contacts |
| 12. Some health workers insulted me and my contacts due to my personal attributes. % DISAGREEING (R) ^c,e,f^ | 121 (98.4%) | 213 (98.2%) | Not applicable to contacts | Not applicable to contacts |
| ***Empowering Care*** |  |  |  |  |
| 13. I was encouraged to select the contact tracing methods that I was comfortable with.^c^ | 115 (93.5%) | 192 (88.5%) | Not applicable to contacts | Not applicable to contacts |
| 14. I was given control over decisions about my contacts. ^c^ | 113 (91.9%) | 186 (85.7%) | Not applicable to contacts | Not applicable to contacts |
| 15. I felt I was given information to understand my choices for notifying network members. ^c^ | 113 (91.9%) | 188 (86.6%) | Not applicable to contacts | Not applicable to contacts |
| 16. I received information and tools to help me protect my family and partners. ^c^ | 95 (77.2%) | 182 (83.9%) | Not applicable to contacts | Not applicable to contacts |

a 1 Index client missing in STANDARD arm

b 15 Index client missing (5 enhanced & 10 STANDARD)

c 1 Index client missing in Enhanced arm

d 16 Index client missing (6 enhanced & 10 STANDARD)

e 179 Contact client missing (63 enhanced & 116 STANDARD

f (R)= Reversed coded
